# The Present and Future Applications of Modern Technologies as Useful Tools in the Management of Cancer: A Narrative Review

**DOI:** 10.7759/cureus.109692

**Published:** 2026-05-26

**Authors:** Victor C Okebalama, Onyebuchukwu Eke, Nwora Oli, Oluyemisi Okwudishu, Obioma Aririsukwu, Cyprian Okoronkwo, Orevaoghene Edewor, Precious Michael, Odutola Odugbemi, Cyril Akwuruoha, David Akwuruoha, Olabiyi Olaniran, Joshua Odeyinka, Christain Ugochukwu, Peace Ogbodo, Chisom Nnadozie, Uzor Victor

**Affiliations:** 1 Department of Anatomic Pathology and Forensic Medicine, Babcock University Teaching Hospital, Ilishan-Remo, NGA; 2 Oncology/Toxicology, Nigeria Police Directorate of Medical Services, Abuja, NGA; 3 Department of Pharmacology and Therapeutics, Ebonyi State University, Abakaliki, NGA; 4 Paediatrics, Nottingham University Hospitals National Health Service (NHS) Trust, Nottingham, GBR; 5 Department of Internal Medicine, Babcock University Teaching Hospital, Ilishan-Remo, NGA; 6 Medical Department, St. Francois Medical Centre, Abuja, NGA; 7 Department of Internal Medicine, King Fahad Specialist Hospital, Tabuk, SAU; 8 Critical Care Unit, Newham University Hospital, London, GBR; 9 Department of Oncology and Palliative Medicine, Jazan Specialist Hospital, Jazan, SAU; 10 Department of Obstetrics and Gynaecology, Babcock University Teaching Hospital, Ilishan-Remo, NGA; 11 Department of Obstetrics and Gynecology, Rivers State University Teaching Hospital, Port Harcourt, NGA; 12 Department of Internal Medicine, Benjamin S. Carson (Snr) College of Health and Medical Sciences, Babcock University, Ilishan-Remo, NGA; 13 Vascular and Endovascular Surgery Department, Beth Israel Deaconess Medical Center, Harvard Medical School Teaching Hospital, Boston, USA; 14 Department of Surgery, Babcock University Teaching Hospital, Ilishan-Remo, NGA; 15 Department of Paediatrics, Babcock University Teaching Hospital, Ilishan-Remo, NGA; 16 Department of Internal Medicine, Novena University, Ogume, NGA

**Keywords:** artificial intelligence (ai), genomic technology, healthcare technology, internet of medical things, machine learning (ml), nanotechnology in medicine, oncology, telemedicine (tm)

## Abstract

Cancer care is becoming increasingly complex globally, with rising cases and challenges for conventional systems. Technology aids in managing this complexity, but it does not replace clinical expertise. The integration of digital tools, artificial intelligence (AI) in imaging and diagnostics, and machine learning in treatment planning marks a shift in oncology. However, successful implementation requires careful evaluation, particularly in nursing practice, where AI assists in symptom tracking and care coordination. Challenges include digital literacy among older patients and ethical issues such as data protection and algorithmic transparency. Rapid adoption without adequate understanding may contribute to long-term negative outcomes. The narrative review explores the present and future applications of modern technology, such as artificial intelligence, robotics, machine learning, telemedicine (including remote patient monitoring), the internet of medical things (such as wearable devices), genomic technologies, and nanotechnology in medicine, among others, in the management of cancer.

## Introduction and background

Cancer quietly grows its weight across the world, rising slowly but steadily, with more cases each year, more lives touched, more families involved, and the systems that once coped seem stretched [1]. It begins with the numbers, the statistics that track incidence and mortality, and then it spreads into experience, into what clinicians see each day, the patients who survive but live with new complications [2]. The old ways, the conventional systems, face strain and struggle to keep up with data, multidisciplinary needs, the different forms cancers take, and the unpredictable ways they present [1]. Clinicians observe this; they notice that human judgment and experience remain anchors, even as new digital tools enter the field: electronic records, imaging platforms, and decision support software, each offering a hand but requiring skill to use [2]. The work of oncology is no longer linear; it involves multiple specialists, teams coordinating across sites, and different perspectives that need integration, with a constant flow of information that is sometimes overwhelming [1]. Technology helps; it extends capacity, it allows patterns to be recognized faster, and it allows decisions to be made with more confidence [2], but it does not replace the clinician; it amplifies, it supports, and it interacts with judgment built over years [1]. The complexity is compounded by the very tools that aim to simplify: training is needed, workflows must adapt, data streams must be interpreted, and yet each small improvement offers a glimpse of control over what feels increasingly unmanageable [2]. Conventional models that relied on memory, manual notes, and episodic consultations feel inadequate, too slow, and too narrow, and this tension between old and new shows why cancer care is not just about the disease but about how the system organizes itself [1,2]. Human skill, judgment, technology, data, and collaboration all swirl together, overlapping, sometimes messy, sometimes repetitive, and yet slowly forming a path toward managing complexity better, toward balancing what is known and what must be discovered, and toward keeping the patient at the center of all these moving parts [1,2].

The scope of modern oncology technology extends beyond advanced medical devices to encompass the wider digital infrastructure that supports cancer care. This includes artificial intelligence systems, digital imaging systems, electronic health records, telehealth services, molecular diagnostics, and data-driven clinical decision support tools that are shaping how cancer is diagnosed, monitored, and treated [1-3]. Some of these technologies are already integrated into clinical practice, while others remain under active evaluation or early development. Understanding this distinction becomes important when discussing both the benefits and the limitations of technological advances in oncology care [3].

Technology did not suddenly redefine oncology. It edged its way in. At first, it appeared as digital record systems, structured databases, and platforms meant to organize information that once lived in paper charts and separate files. That shift seemed modest, almost logistical, yet it quietly changed how clinicians accessed and shared patient data [3]. Once information became easier to retrieve and connect, other possibilities began to surface. Artificial intelligence entered through imaging analysis and diagnostic support, offering pattern recognition that extended beyond what the human eye could consistently detect [4]. Machine learning models followed suit, drawing on accumulated cases, refining predictions over time, and gradually embedding themselves into treatment-planning discussions [5]. Still, none of these systems stood alone. They required clinical framing, contextual understanding, and interpretation shaped by experience [3]. As these tools moved into radiology, pathology, and therapeutic decision systems, oncology practice began to feel more interconnected, more data-driven, and perhaps more complex [6]. Automation reduced repetitive analytical burdens, algorithmic systems generated probabilities, and predictive tools became part of multidisciplinary conversations [4,5]. Yet something remained constant. Clinicians still questioned outputs, weighed uncertainties, and adjusted recommendations based on patient-specific realities [6]. Artificial intelligence could assist, sometimes impressively, but it did not replace judgment. Instead, it introduced a new layer of dialogue between human reasoning and computational suggestion [4].

Studies on breast cancer screening indicate that AI-supported mammography systems can enhance cancer detection rates while reducing some of the repetitive burden placed on radiologists during screening. Some evaluations and population-based implementation studies suggest that AI-supported mammography systems can be effectively integrated as supplementary tools within existing screening workflows, particularly in high-volume settings where radiologists must interpret large volumes of imaging data [7,8].

Similar applications have been seen in digital pathology, where AI-assisted systems have aided in tumor detection and slide interpretation, particularly in settings managing increasing diagnostic volumes and increasingly complex image datasets [9]. Systematic reviews of diagnostic accuracy in digital pathology reports show promising results across multiple tasks. Nevertheless, most systems require further validation across diverse institutions and patient populations before standardization for broad clinical use becomes feasible [9]. Despite these advances, physicians continued to review AI-generated outputs carefully, especially in cases that were ambiguous or deviated from typical presentations [7,9]. While the technology contributed to improved processing speed, consistency, and analytical capacity, interpretation ultimately depended on human expertise, clinical experience, and multidisciplinary evaluation. Over time, this interaction settled into everyday practice. What once felt novel became embedded. And oncology began to operate in a blended space where digital systems, analytic intelligence, and human expertise intersect continuously, shaping diagnosis, workflow, and treatment decisions together [3,6].

It is one thing to introduce technology into cancer care, and another to understand what it is truly doing. Artificial intelligence in cancer treatment has shown measurable promise, particularly among older adults, for whom treatment decisions are often more delicate and layered. Yet the same review that reports benefits also reveals inconsistency, variation in outcomes, and areas where evidence remains limited. That contrast is difficult to ignore. If results differ across populations, then careful examination becomes necessary. Not dramatic examination, not excitement-driven adoption, but structured and patient review [10]. In oncology nursing practice, artificial intelligence tools are beginning to assist with symptom tracking, care coordination, and decision support [11]. But even there, integration depends on workflow, training, ethical clarity, and system readiness [11]. These conditions are not equal everywhere. Older patients may face barriers related to digital literacy or access, and nurses may work within systems that are not fully prepared for technological layering [10,11]. When these realities are placed beside technological optimism, a tension quietly forms. The question shifts from what technology can do to who it truly serves. Ethical concerns follow naturally. Data protection, algorithm transparency, bias within training datasets, and the risk of overreliance on automated recommendations are not distant worries; they are present considerations [11]. And when vulnerable groups are involved, those considerations weigh more heavily. This is why examining present applications is not enough. There must also be a forward-looking evaluation, a deliberate effort to question sustainability, equity, and long-term clinical impact [10]. Without structured reflection, adoption may outpace understanding. And in cancer care, where decisions carry weight beyond the immediate moment, that imbalance can quietly shape outcomes for years to come [10,11].

Methods 

During the course of eleven months (from May 1st, 2025, to March 31st, 2026), a thorough literature review was conducted utilizing the databases Google Scholar, the Cochrane Library, PubMed, ScienceDirect, and Scopus. Amalgamations of keywords such as "healthcare technology", "oncology", "modern technology in oncology", "artificial intelligence", "machine learning", "telemedicine", "nanotechnology", "internet of medical things", and "genomic technology" were used to find pertinent publications authored in English. The selection of studies was based on their applicability to current and emerging uses of contemporary technology in the treatment of cancer. Four hundred and twenty-five research articles were produced from the initial investigation. This was reduced to 67 articles.

In selecting the final research articles, studies that were not peer-reviewed and with no clear or prospective applications in humans with oncologies were excluded, while studies that were peer-reviewed and with current, experimental, and/or prospective applications in the clinical oncology care of humans were included. Additionally, in synthesizing the evidence narratively, studies were grouped by technology type, current clinical applications, and experimental or prospective clinical applications in oncological care in humans, emphasizing domains of convergence, disagreement, and voids in the literature while incorporating data from other research and spotting trends.

## Review

Conceptual foundations of modern technology in oncology

When we say modern technology in oncology, it is easy to imagine equipment, machines, something visible and mechanical. But that is not really what the term settles on when examined closely. It is less about hardware and more about digital structure. More about systems that hold information, move information, and make information usable within cancer care [12]. Digital health sits at the center of that definition. It includes electronic health records, remote monitoring tools, patient communication platforms, and telehealth systems, all of which reshape how patients and clinicians interact across time and distance. And underneath these visible layers, there are data systems quietly operating, collecting, organizing, integrating, and translating large volumes of clinical data into something coherent. Artificial intelligence then becomes a function within that environment, not separate from it, but dependent on it, drawing from structured datasets to assist with interpretation and decision support. The boundaries begin to form here. Modern technology in oncology does not mean every innovation or every advanced device. It refers specifically to this digital and data-driven ecosystem that supports diagnosis, treatment planning, follow-up, and system evaluation. It is guided by frameworks that emphasize interoperability, patient-centered design, ethical governance, and coordinated implementation across care settings. And when viewed this way, the term feels less abstract. It becomes clearer that modern technology is not a collection of tools scattered across departments. It is an organized digital architecture that now underpins how oncology functions, often quietly, often in the background, but consistently shaping the way care is delivered and understood [12].

If we think back to oncology before the digital wave, it was not primitive, but contained. Tools were physical, imaging devices, laboratory instruments, paper charts stacked in files, and much depended on trained eyes and accumulated experience. Technology existed, certainly, but it did not speak to itself. Information moved slowly, often manually, and decisions were gathered around meetings, discussions, and interpretation. Then something subtle began to change. Records became electronic, images became digitized, and systems began storing data in ways that allowed retrieval beyond memory alone [13]. At first, this felt administrative, almost logistical. Yet over time, that shift altered how cancer care functioned, because once information could flow more freely, patterns became easier to trace. The emergence of artificial intelligence marked a deeper turning point. Instead of simply holding information, systems began to examine it, identifying structures within data that were not immediately visible to clinicians. Algorithms entered imaging, diagnostics, and even treatment planning, not replacing expertise but expanding analytical reach [14]. Around what is often described as the fourth industrial revolution, this layering of digital systems and intelligent computation accelerated. Healthcare professionals began to sense that the change was no longer incremental but structural, that cancer care was moving toward interconnected platforms rather than isolated tools [13]. Computational power increased, datasets expanded, and research into AI applications in diagnosis and treatment intensified. Looking across this progression, the evolution does not appear sudden. It feels cumulative. A steady movement from physical instruments, to digital storage, to analytic intelligence, each stage building quietly on the last, until oncology found itself operating within a technological environment far more integrated than it once imagined [13,14].

When technology settles into oncology, it does not choose one corner of practice and remain there. It spreads. A telehealth system introduced for remote consultation begins to influence nursing workflows, physician scheduling, radiologic follow-up, and even how laboratory results are reviewed [15]. What appears at first as a communication tool gradually reveals layers beneath it. There are platforms that must integrate records, engineers who maintain system stability, and data structures that allow information to move without distortion [16]. Radiologists read images that are no longer confined to physical films but embedded within shared digital environments. Pathologists review specimens within systems that connect directly to broader clinical data. Nurses monitor symptoms remotely and coordinate care through interfaces that connect multiple professionals simultaneously [15]. And behind each of these interactions are technical teams shaping interoperability, ensuring security, refining usability [16]. The more one looks, the less isolated each role appears. Digital health in cancer care seems to blur traditional boundaries, not by removing them, but by weaving them together through shared systems [15]. Remote oncology care makes this weaving more visible because distance forces clarity in coordination and exposes any weakness in communication pathways. What emerges is not technology sitting alongside clinical work, but a cross-functional digital ecosystem in which oncologists, nurses, radiologists, data scientists, and engineers depend on one another to sustain continuity of care [15,16]. The integration feels less like an addition and more like a restructuring, gradual yet profound, altering how disciplines interact, how decisions circulate, and how responsibility is shared within modern oncology practice [16].

Data in oncology were once charts, laboratory values, and imaging reports, filed and revisited when needed [17]. Useful, but passive. Now the volume alone has changed things. Clinical records, genomic information, imaging archives, and treatment outcomes all accumulate across systems and institutions, forming what is called big data environments [18]. Decisions are increasingly shaped by aggregated patterns rather than isolated cases [17]. Real-world evidence reflects not only trial conditions but also everyday clinical realities, diverse patients, varied responses, and long-term follow-up. Artificial intelligence systems depend on this structure because their ability to recognize patterns rests entirely on the depth and diversity of the datasets that trained them [17]. Patient-generated health data adds another dimension. Remote monitoring tools, wearable devices, and symptom reporting platforms contribute continuous streams of information beyond clinic walls [18]. Data begins to resemble a form of clinical currency. It circulates through systems, informs decision support platforms, and guides treatment planning [17]. Yet its value depends on interoperability, governance, and careful digital infrastructure [18].

Technology in oncology brings excitement, but it also brings hesitation. Healthcare professionals recognize progress, yet they express uncertainty about whether systems are ready [13]. Artificial intelligence promises efficiency and precision, but questions arise. Are institutions actually prepared to integrate these tools responsibly [17]? Access becomes a concern early. Digital health platforms and telehealth can extend cancer care beyond hospitals and clinics, but they require stable internet access, technological familiarity, and infrastructure that supports them [15]. Not every patient population has these conditions. In that gap, something becomes visible, a divide.

That divide becomes even more visible in low-resource settings, where the promise of tele-oncology frequently meets practical limitations that cannot be overlooked. In some regions, internet connectivity remains unstable, electricity supply is unreliable, and access to digital devices is uneven across communities [18,19]. A remote consultation platform may be available, yet its sustained use is often undermined when patients lack consistent online access or when healthcare facilities lack the infrastructure required for secure digital communication [15]. Financial barriers further complicate implementation. Reimbursement structures for telehealth services remain inconsistently defined, particularly in developing healthcare systems where digital oncology models are still emerging [18]. Beyond infrastructure and funding, digital literacy presents a less visible but significant barrier. Older adults and individuals with limited exposure to technology often encounter difficulty navigating virtual systems, while healthcare professionals may also require additional training to adapt to these digital workflows [15,19]. Consequently, what initially appears as a technological intervention reveals underlying structural inequalities within healthcare systems. The challenge shifts from deploying tele-oncology platforms to ensuring that institutions and communities are equally positioned to benefit from them.

This distinction between the availability of a technology and its practical accessibility becomes increasingly important when evaluating the adoption of digital oncology tools across diverse healthcare settings [19]. Implementation is rarely smooth. New systems require workflow changes, training, financial investment, and coordination across departments [17]. Without these supports, adoption stays fragmented and incomplete. Regulatory oversight struggles to keep up, especially around data governance, algorithm validation, and accountability when automated systems influence clinical decisions [13]. Ethical questions deepen the conversation. Bias in datasets, limited transparency in how algorithms are built, and uncertainty about who holds clinical responsibility when something goes wrong [17]. These raise real concerns about fairness, about trust. Workforce adaptation is another layer. Clinicians must interpret outputs they did not create, nurses navigate remote care platforms, and institutions must build digital skills that go beyond traditional medical training [15]. Technological adoption looks less like an upgrade, more like a transformation [13,17].

Artificial intelligence and machine learning in oncology

Artificial intelligence enters the biological space of oncology through learning models that try to interpret complexity. Supervised learning models start with labeled datasets where outcomes are already known [20]. Algorithms learn to associate molecular patterns or clinical features with specific cancer types. Unsupervised models move differently. They explore datasets without predefined labels, searching for hidden structures, clustering genomic signatures, and identifying subtypes that may not yet be clinically defined. Deep learning expands this further. Neural networks, structured in layers, process high-dimensional biological data, extracting relationships across imaging, genomic, and transcriptomic inputs. This becomes relevant because cancer is not driven by a single mutation or pathway, but by interacting biological systems. Multi-omics integration attempts to bring together genomics, proteomics, transcriptomics, and other molecular layers into a unified framework. The challenge is not simply gathering data, but interpreting it biologically. Learning models are tasked with recognizing patterns reflecting tumor heterogeneity, treatment-response variability, and disease-progression mechanisms. Neural networks may detect correlations among molecular features that would be difficult to trace with conventional approaches. Yet these models do not replace biological reasoning. They depend on structured datasets, careful validation, and domain expertise to ensure computational patterns correspond to meaningful biological processes. Algorithmic learning becomes less about automation, more about discovery. Importantly, many multi-omics and deep learning applications remain concentrated within translational research environments and specialized academic institutions. Wider clinical adoption continues to be constrained by the need for further validation, requirements for computational infrastructure, and the demands of regulatory oversight [20].

Artificial intelligence is slowly becoming a companion in the diagnostic process, quietly analyzing images that once required constant human scrutiny [6]. In radiology, scans are no longer just read; patterns are detected, subtle changes are highlighted, nodules are measured, and features are quantified. Pathology slides, once examined entirely by eye and experience, are now examined with digital assistance [21]. AI learns from thousands of images, recognizing structures, textures, and cellular arrangements, sometimes suggesting what might have been overlooked [21]. Radiomics and predictive models extend this further, taking the invisible and making it meaningful [6]. Early detection systems serve as second opinions, continuously learning and adjusting, helping clinicians catch tumors before they grow or spread [21]. It is not about replacement, it is about support, a layering of human judgment and machine insight. The promise is profound yet uneven, and each success underscores that technology, while powerful, reaches its potential only when paired with human expertise, careful validation, and thoughtful integration [6,21].

Predictive analytics in oncology is reshaping how clinicians think about what might happen next. Models sift through patient histories, laboratory values, imaging results, and genomic data, searching for patterns that might escape immediate notice [22]. Risk stratification no longer relies solely on clinical intuition. Algorithms weigh variables across cases, highlighting patients who might deteriorate faster, those who might respond better to certain treatments [23]. Survival prediction models emerge from data layers that are nearly impossible for humans to process fully, yet they still require judgment, context, and the careful eye of a clinician to make sense of what the numbers suggest [22]. Treatment response forecasting is becoming more precise over time. Therapy selection can now be guided by probabilities and correlations buried deep in data that would be difficult to identify manually [23].

Several precision oncology approaches are already demonstrating measurable clinical benefit, although the strength of evidence varies across different settings and cancer types. Biomarker-driven therapies and genomic profiling platforms have improved treatment matching in certain cancers, particularly where specific molecular alterations can directly inform therapeutic choice [24]. In some cases, this means extended progression-free survival and more individualized treatment pathways, especially within targeted therapy and immunotherapy contexts [24,25].

However, the broader picture is more complex than the initial optimism implied. Comprehensive genomic testing remains costly, and the cost-effectiveness of precision oncology depends heavily on local healthcare infrastructure, reimbursement systems, and the availability of targeted treatments [26]. Access is also unequal. High-income countries generally have the laboratory infrastructure, sequencing technology, and specialist expertise required for implementation, while lower-resource settings often face barriers related to affordability, testing availability, and integration into routine clinical care [25]. So, while predictive analytics and precision medicine continue to reshape oncology, their advantages are not evenly distributed. Although several biomarker-guided therapies have been adopted into international oncology guidelines, many predictive AI-driven treatment models are still undergoing evaluation and have not yet been fully standardized in routine clinical practice [22-24].

The process is iterative. It is never perfect. Models learn from each new case, looping back, adjusting predictions, refining their outputs. Sometimes they surprise. Sometimes they confirm what was already suspected. Through all of this, the promise remains clear. Clinicians are supported in their decisions, choices become more informed, and patients may be spared unnecessary harm. But the human mind stays central, translating numbers into meaning, into actual choices, into care that feels personal [22,23].

Artificial intelligence in cancer care is powerful, but it carries quite a few imperfections. The datasets it relies on are not always representative. Sometimes they reflect only certain populations, leaving blind spots in others [22]. Small differences in genetics, lifestyle, or local healthcare practices can lead to outcomes that differ from the model's expectations [6]. Overfitting is another subtle risk. Models can appear confident but become over-tuned to past data, struggling when they encounter something slightly different [22]. Validation gaps mean that even promising predictions need repeated testing, careful checking, and continuous oversight before they can be trusted in practice. Ethical concerns run through this work. Biased predictions can reinforce inequalities, misguide treatment decisions, and create harms that were not anticipated [6,22]. Regulatory frameworks seek to guide this rapidly evolving field, but rules are often reactive, uneven, and lag behind the pace of innovation [23]. Clinicians must navigate this carefully. They learn to read AI outputs skeptically, revisit anomalies, question suggestions, and integrate algorithmic insights with experience, intuition, and the context of each patient's life. The technology can highlight patterns, suggest probabilities, and speed up analysis. But it cannot feel the weight of a patient's circumstances. It cannot weigh values. It cannot replace judgment. AI challenges the human mind to think differently, to check assumptions, and to decide with care. And it is in that interplay between machines and humans that real progress in oncology emerges [6,22,23].

Intelligent clinical decision support is changing how oncologists approach their work. These systems analyze patient data, genetic profiles, lab results, imaging, and treatment histories, searching for patterns that might not be immediately visible [20]. They propose options, suggest risks, and sometimes point toward possibilities that were not obvious at first [22]. Integration into workflows has been gradual. Some institutions have adopted these tools quickly, others more cautiously, still learning how algorithmic output fits alongside clinical judgment [23]. Precision oncology platforms can suggest tailored therapies based on molecular profiles, flag potential adverse responses before they happen, and support decisions about prognosis in ways that feel more grounded in data [20]. But caution remains. Models continue to evolve, datasets grow larger and more diverse, and predictions need to be validated against what actually happens in practice [22,23]. Ethical questions do not disappear. Patient circumstances still matter. The clinician still interprets what the system offers, weighing it against experience, against intuition, against what they know about the person in front of them [20,22,23]. This reveals that clinical decision support systems primarily serve as assistive tools rather than independent decision-makers. Their reliability still depends on clinician oversight, institutional governance, and continued validation across diverse healthcare environments [22,23]. Machine learning tools may become more sophisticated over time, their suggestions more refined, their integration more seamless. Yet the core of oncology care remains human. AI informs, supports, and expands what is possible to see. But the decision, the conversation, and responsibility stay with the people providing care [20,22,23].

Genomic technologies and precision oncology

Tumors are rarely simple. Each one evolves differently, accumulates mutations over time, and shifts in behavior as it grows [27]. Molecular profiling tries to make sense of this complexity, moving through data layer by layer, piece by piece [28]. Sometimes a mutation stands out clearly. Other times, it is subtle, barely detectable, and clinicians have to decide what matters, what might be actionable, what could just be noise [27,28]. Understanding these patterns takes time. It depends on repeated observation and on linking what the data shows to what actually happens in patients. Technology can reveal hidden patterns and illuminate what was previously invisible. But judgment is still needed. Experience is still needed. Context is still needed to translate what the profile shows into decisions about care. Molecular profiling opens a window into tumor heterogeneity. It shows the differences that exist within a single tumor, between tumors, and across patients. Yet the human mind remains essential, returning to the same data, checking again, reconsidering, making sense of patterns as they slowly emerge. Many of these molecular profiling approaches have become part of standard oncology practice, especially for cancers in which specific genomic alterations inform the choice of targeted therapy. In contrast, the clinical value of broader profiling remains less well established, particularly for tumors with unclear mutations or for which treatment is still emerging [27,28].

Next-generation sequencing generates streams of genetic data. It can suggest therapies based on specific mutations, reveal why resistance might develop, and hint at what treatment might work best [29]. But it does not answer questions on its own. Clinicians sift through the mutations that appear, decide which ones are significant, which can actually guide therapy, and which require caution before acting. Some patients respond exactly as the sequencing predicted. Others do not. And this keeps the process reflective, requires returning to the data, thinking through what went differently. Sequencing is powerful, but its value grows slowly. It grows through dialogue with human interpretation, through discussion across clinical teams, through repeated analysis and reconsideration of what the data means. The technology expands what is possible, challenges what was assumed. But it always requires judgment, patience, and careful attention to the context of each patient's situation. Current sequencing technologies are used to identify targetable mutations and mechanisms of resistance. However, interpreting the results is challenging. A substantial proportion of the detected variants are of uncertain clinical significance [29]

Biomarkers act as signposts. They tell clinicians where to look, which therapies might succeed, and which might fail [27,30]. But they are never the full story. Companion diagnostics translate these signs into actionable decisions, but the process is not straightforward. A test might suggest a particular therapy, but context still matters. Patient age matters. Tumor behavior matters. Other health conditions matter. All of these influence what decision gets made. Human judgment loops back on itself constantly. Test, decide, observe the outcome, and adjust based on the outcome. Technology can reveal possibilities that were not visible before. But outcomes are shaped by how the clinician interprets what the test shows, by their experience with similar cases, and by repeated reflection on what works and what does not. Oncology becomes a conversation between data, tools, and people. Each case teaches something. Each reinforces or challenges what was believed. Each reminds us that the work is both technical and deeply human. A number of biomarker-driven therapies are now approved and built into oncology guidelines for cancers like lung, breast, colorectal, and blood cancers. But the way these biomarkers perform varies depending on the population and the tumor types. As a result, validation and interpretation remain ongoing processes rather than fixed endpoints [27,30].

Even when sequencing technologies exist, they encounter reality. Hospitals differ in resources, in staff expertise, in what they can actually access [28]. Some have the infrastructure to implement precision approaches smoothly, others do not [29]. Pediatric oncology shows these disparities clearly. Precision therapies exist for certain childhood cancers, but access is uneven, implementation is challenging, and outcomes depend heavily on where a child receives care [31]. Economics shape what is possible. Workflow constraints shape it. Reimbursement policies shape it. Regulatory frameworks shape it. All of these slow the path from discovering a treatment approach to actually delivering it to patients [28,29,31]. Clinicians negotiate these barriers continuously, balancing what is ideal against what is actually feasible in their setting. Some solutions succeed quickly. Others require iteration, adaptation, patience, and a willingness to try again in different ways. It is never straightforward, but progress happens incrementally, shaped by the people working within these constraints [28,29,31].

Looking forward, gene editing and AI-powered genomic analysis hint at something different. Therapies tailored not only to tumor type but to each patient's specific molecular profile. Predicting how someone will respond before treatment even begins. These possibilities feel closer now [27,32]. Clustered regularly interspaced short palindromic repeats (CRISPR) and base-editing technologies promise the ability to correct mutations at their source and to intervene at the genetic level in ways previously impossible [32]. AI systems integrate data from genomics, proteomics, transcriptomics, and other molecular layers to suggest optimal strategies based on patterns across thousands of cases [32]. Yet this potential is tempered. Ethics raises questions. Regulation moves cautiously. Validation takes time. Each advance must be tested carefully before it can be trusted in practice [27,31,32]. The future feels near and distant at once. Each step forward illuminates new possibilities but also reminds us that judgment, experience, and careful implementation remain central to how these tools will actually be used. Progress will continue, but it will be iterative, reflective, and shaped fundamentally by the people guiding it. At present, many AI-driven genomic prediction systems and gene-editing approaches remain within translational research or early clinical investigation rather than routine oncology care [32]. While early findings look promising, implementing widely isn’t that simple. Before it can be adopted at scale, we need to consider long-term safety evaluation, reproducibility across diverse populations, regulatory oversight, and prospective clinical validation [27,31,32].

Advanced imaging and diagnostic innovations

Radiologic imaging did not simply improve over time. It matured. And as it matured, it changed how oncology thinks [33]. There was a time when seeing a tumor meant identifying its shape and size, drawing margins around it, and measuring its diameter. That felt sufficient [33]. But sufficiency is rarely enough in cancer care. Positron Emission Tomography-Computed Tomography (PET-CT) changed things by adding metabolic activity to anatomical structure. Suddenly, a mass could appear small yet burn intensely due to metabolic uptake. Or it could appear large but remain biologically quiet [33]. That difference forced clinicians to reconsider what they were actually seeing. Shape alone was not enough anymore. Then other innovations arrived. Multiparametric imaging techniques began pulling out texture, perfusion, how contrast behaved over time, details that had always been there but were now visible [34]. In precision radiation oncology, imaging stopped just showing where the tumor was. It started guiding where the radiation should go, how the dose should be shaped, and how therapy could adapt based on what the biology was doing rather than just geometry [35]. Even with all this, clinicians still return to the scans repeatedly. They compare across time, question what looks unusual, and reconsider what they thought before [33-35]. Imaging evolves through technology, yes. But it also evolves in the way it reshapes how oncologists think, reason about what they see, and sometimes pause before deciding. Imaging technologies like PET-CT and image-guided radiotherapy are already standard components of oncology. However, more advanced approaches, such as multiparametric and biologically adaptive imaging strategies, are still being refined and validated more broadly before they can be adopted [34,35].

At some point, oncology realized tumors are not isolated objects. They are ecosystems. And that realization changed what imaging needed to show [36]. Structural clarity was not enough anymore. Clinicians wanted to see oxygenation, how blood vessels were working, where immune cells were gathering, and how metabolism was shifting. Functional imaging techniques, especially advanced magnetic resonance and optical approaches, started capturing these signals. Things invisible to the naked eye became visible. Hypoxic zones could be mapped. Perfusion irregularities could be seen. Metabolic shifts could be tracked as they happened over time [36]. But making sense of this is rarely simple. A hypoxic region might suggest the tumor will resist radiation. But then its relationship to blood supply complicates that picture [29]. An imaging signal might change from one scan to the next, and clinicians have to ask whether they are watching the tumor respond to treatment or adapt in some other way [36]. Functional imaging gives deeper insight. But it also brings deeper uncertainty, because the more detail you see, the more complexity emerges. The mind has to circle back. Check against symptoms. Check against lab values, earlier imaging and then pause, reassess and finally decide. What comes out of this is not just a clearer image. It is a more dynamic sense of the tumor as something living, something that adapts [36]. Functional imaging techniques are playing a bigger role in oncology research and in some specialized clinical settings, but a lot of their use is still at the translational stage. It has not been standardized enough yet to be part of routine care across all cancer types. Interpreting these scans also takes real expertise. What shows up can change based on the tumor microenvironment, when the scan is done relative to treatment and the specific imaging method used [36].

The idea that cancer could be monitored through blood once felt speculative. Almost too simple. Yet circulating tumor DNA has slowly redefined how surveillance works. It introduced a way to check the molecular pulse of disease through routine blood draws [37]. Tiny fragments of genetic material circulate quietly in the bloodstream. They carry signals such as mutations, resistance patterns and sometimes signs of relapse before anything shows up on imaging. The appeal is clear. It is minimally invasive. It can be repeated easily. It allows tracking of how the disease evolves without going back for tissue samples each time [37]. But the enthusiasm has to be careful, low levels of DNA fragments require very sensitive tests. Background noise can interfere. The challenge intensifies in patients with minimal tumor burden, in whom circulating DNA fragments may be insufficient for consistent detection [38]. In such cases, a negative result does not necessarily indicate the absence of disease; the molecular signal may simply fall below the reliable detection threshold of the assay.

Variability across assays introduces further complexity. Different laboratories use distinct sequencing methods, thresholds, or reporting standards, which can lead to discrepancies in results interpretations across institutions [38,39]. Clinicians, therefore, have to think carefully about what the reported numbers actually represent. A molecular signal detected under a set of conditions may not be detected, or may be reported differently, in another. And because ctDNA technologies are still evolving, standardization remains uneven, particularly as these tests move from controlled research environments into routine clinical use [39].

And not every mutation that gets detected actually means a treatment change is needed [37]. Clinicians find themselves layering evidence. Comparing what the ctDNA shows with what imaging looks like, with what the patient is experiencing, and with what earlier genomic tests revealed. The process is reflective. It is iterative. It is not about reacting immediately. In pancreatic cancer and other aggressive types, liquid biopsy offers an early warning. But the signals need context. They need validation. Sometimes they need to be watched over time before anyone acts. It is promising. Deeply promising. But it asks for restraint, for discipline, for checking again before moving forward [37]. ctDNA liquid biopsies are already making their way into certain parts of oncology, especially for molecular profiling and monitoring treatment response in some advanced cancers [38,39]. Using them more broadly for early detection and routine follow-up is trickier. The main challenges are assay sensitivity, lack of standardization, cost, and the need for prospective studies that hold up across different clinical settings [37-39].

For decades, pathology happened around the microscope and the trained eye. Digital pathology disrupts that. It converts glass slides into images that computers can examine [40]. Algorithms can count mitotic figures, detect architectural distortions, analyze patterns in the stroma, and even link what they see under the microscope with genomic data and imaging from other sources. This expands consistency. It reduces variability caused by human fatigue or differences in training. But it does not remove the human part. In practice, the work becomes cyclical. The algorithm points something out. The pathologist reviews it. When there is a mismatch, they reassess. The feedback is used to refine the model. Multimodal learning systems take this further. They integrate pathology into the broader picture of oncology, connecting what is seen at the microscopic level with what shows up on scans and what appears in molecular tests. What makes this transformation meaningful is not automation by itself. It is amplification. The machine finds patterns across thousands of cases. The human expert brings context, interpretation, and final judgment. The outcome is not replacement; it is a collaboration and it keeps evolving. Digital pathology adoption continues to expand across oncology centers, especially where the digital infrastructure and computational pathology programs are already established. But implementation remains uneven globally; regulatory approval, interoperability between systems, storage demands, and the need to validate across different lab settings are all slowing down how quickly it gets implemented more broadly [40].

What marks this era is not one imaging technique becoming better. It is different techniques being brought together. Imaging data now flows into precision oncology platforms. These platforms combine imaging findings with genomic patterns, treatment histories, and clinical variables that are updated in real time [34,35]. Image fusion technologies layer different scans on top of each other, creating composite views that help guide decisions right there in the moment [41]. Adaptive radiation strategies depend on this. They adjust radiation doses based on biological feedback instead of following a fixed plan set weeks earlier [35]. But bringing everything together is not smooth. Workflows have to be redesigned. Data standards have to be made compatible. Clinicians have to learn how to read outputs that combine multiple sources [34,35]. Trust builds slowly, through studies that validate the approach, through cases where it works and through careful review when it does not [41]. The process keeps looping. Imaging informs what therapy to use. Therapy changes how imaging gets interpreted. AI systems take in the evolving data and update their suggestions [34,41]. Precision oncology is not a fixed point to reach. It is a process, iterative and reflective. Each imaging method adds one piece to a larger picture. And at the center sits the human clinician, filtering, questioning and weighing what the probabilities say against what experience suggests. Making sure the technology serves the patient instead of becoming overwhelming. The shift toward digital care can be clearly seen in integrated imaging platforms and adaptive oncology systems. But the problem is, many of these workflows are still developing, and outside of a handful of highly specialized cancer centers, they haven’t become routine practice [34,35,41].

Technological innovations in cancer treatment modalities

Surgical oncology has been quietly transformed by robotic-assisted platforms. Surgeons navigate instruments with a level of precision that was not possible before [42]. Yet the learning curve is gradual. Sometimes uneven. Some adopt the technology quickly, others more slowly. Operations that once demanded hours of manual dexterity now integrate robotics to stabilize movements, guide instruments, and amplify what the human hand can do. But judgment remains central. These systems allow complex resections, minimally invasive procedures that expand what is surgically possible. Yet each step depends on human oversight. The technology does not replace intuition; it sits alongside it, offering repeated patterns, visual clarity, and subtle feedback that the surgeon interprets in real time. What develops is a hybrid practice. Human and machine working together, reshaping surgical oncology in ways both subtle and significant. Robotic-assisted surgery is now clinically established in several oncologic procedures, particularly within urologic, gynecologic, and colorectal surgery. However, access to robotic systems remains uneven because implementation depends heavily on institutional funding, surgeon training, maintenance costs, and procedure volume [42].

Radiotherapy has evolved. Image guidance and proton beam therapy offer finer control over how tumors are targeted [42,43]. Patients are positioned, imaged, and treated with an accuracy that once seemed aspirational. Yet each scan, each treatment plan loops back through human review, clinical judgment and verification [43]. The technology allows dose modulation, sparing of healthy tissue in ways that were not possible years ago [42,43]. But it is never purely automated. Clinicians guide the process, adjust parameters, and interpret the feedback as it comes in [43]. It becomes a system of repeated observation and refinement. Errors are minimized, but vigilance remains necessary. Technological innovation meets professional experience, and the balance is delicate. Image-guided radiotherapy has become a routine component of many modern radiation oncology programs. Proton therapy, on the other hand, is still only available in select places because of its high infrastructural and operational costs [42,43]. Ongoing studies continue to evaluate which patient populations benefit the most from it compared to other approaches [43].

Chemotherapy delivery has begun to use nanotechnology for precision. Drugs are carried in nanoparticles designed to release therapeutics at the tumor site [44,45]. Yet each design and each dose calculation is filtered through careful clinical consideration. The potential to improve treatment effectiveness while limiting damage to the rest of the body is significant, but the path from laboratory to patient is slow, iterative, and filled with checks and refinements [45]. Every innovation gets layered onto protocols that are already established. Human oversight sits at the intersection of engineering, pharmacology, and oncology practice. Patients benefit from these improvements as they come, even as the technology continues evolving and is never static, always guided by both science and judgment. While selected nanotechnology-based drug delivery systems have already entered clinical oncology practice, many advanced nanotheranostic platforms are still in the experimental stage and are undergoing translational and early-phase clinical evaluation. Questions regarding their scalability, long-term safety, manufacturing consistency, and cost-effectiveness continue to shape the extent to which these technologies can be implemented [44,45].

Immunotherapy has moved forward with bioengineering interventions. Chimeric Antigen Receptor T-cell (CAR-T) cells, next-generation immune modulators, and engineered delivery systems that offer targeted attacks on tumors. Yet each therapy is individually designed. Monitored. Adjusted based on what happens. The technology is powerful, but it moves alongside clinical expertise. Clinicians evaluate how patients respond, manage side effects as they appear, and iterate protocols when needed [45-47]. Certain side effects associated with CAR-T therapy can escalate rapidly. Cytokine release syndrome remains one of the most closely monitored complications of CAR-T therapy, particularly when immune activation becomes excessive and systemic inflammation intensifies [48]. Early manifestations often include fever and fatigue, but patients can deteriorate quickly into hypotension, respiratory distress, or multi-organ dysfunction if the response is not identified and managed promptly [48].

Neurotoxicity presents an additional source of clinical uncertainty. Manifestations such as confusion, tremor, aphasia, and seizures may appear, sometimes subtly at onset, and require immediate evaluation and intervention [49]. These toxicities do not diminish the therapy value of CAR-T, but they remind clinicians that even highly engineered treatments can be unpredictable. Consequently, monitoring is continuous. Clinical teams reassess patients frequently, weighing therapeutic efficacy against the degree of immune activation [48,49]. Cells programmed to fight disease might seem almost automatic. But they are the product of careful calibration, trial, and observation. Human insight remains central even as machines and engineered biology extend what is possible [45-47]. Regulatory approval has been granted for several CAR-T therapies for specific hematologic malignancies and they are now integrated into specialized oncology practice. Extending their use to solid tumors and to broader healthcare systems, however, remains constrained by challenges in toxicity management, manufacturing complexity, high costs, and the requirement for highly specialized clinical infrastructure [46-49].

Looking ahead, surgical robotics, radiotherapy innovations, and digital control systems are moving toward something more integrated. An oncology ecosystem where different modalities connect. Decisions will loop across these systems. Data-driven but constantly interpreted by humans, aligning precision with judgment. Workflows will become interconnected and adaptive, reflecting a reality where technology amplifies what clinicians can do but does not replace them. The future is iterative and layered, every decision and plan is informed by data but guided by experience, inherently human [42,43,46]. It is a vision of oncology that feels both intricate and grounded. Experimental yet practical. A reflection of how humans navigate complexity with tools that extend their capabilities rather than replace them. Despite ongoing technological advancements, implementation barriers persist. Regulatory processes, interoperability, clinician training requirements, and uneven access across regions continue to determine how quickly these systems can move from specialized settings to broader clinical practice [42-46].

Nanotechnology and targeted therapeutic systems

The promise of nanocarriers in oncology started with a problem that kept coming up. How to deliver potent drugs directly to tumors without overwhelming healthy tissue [50-52]. Conventional chemotherapy spreads broadly through the body. It can work, yes, but the toxicity it causes reflects that lack of precision. The drug does not distinguish well between cancer cells and healthy cells. Nanoparticle systems try to fix this imbalance. They use engineered carriers that alter drug transport through the body, improve their solubility, and extend their circulation before clearance [50,51]. Liposomes, polymeric nanoparticles, dendrimers, and metallic structures. These are not just passive containers holding medication. They are platforms designed to move through biological barriers, to take advantage of how tumors leak and retain materials differently than normal tissue does, and to accumulate preferentially where the tumor is located [50,52]. What becomes clear across the research is that how effective a therapy is depends not only on the drug itself. It depends heavily on the structure of what carries it, on how that carrier behaves in the body [51,52]. The nanocarrier changes where the drug goes, protects it from breaking down too early as it circulates, and reshapes the balance between how much benefit the drug can provide compared to the harm it might cause [50].

Passive accumulation alone is not enough. The research keeps returning to this point. Specificity matters [50,53,54]. Tumor-specific targeting works by attaching molecules to the surface of nanoparticles. Antibodies, peptides, and small molecules that recognize receptors overexpressed on the surface of cancer cells [50,53]. Active targeting tries to turn probability into precision. It guides the therapeutic payload toward specific cell populations while limiting how much reaches other parts of the body [54]. The complexity comes from biological variation. Receptor expression changes not only between different tumor types but within the same tumor mass, some areas express more, some less [50,54]. Targeting strategies have to account for this heterogeneity. They balance how strongly the nanoparticle binds to its target, how stable it remains in circulation, and how efficiently it gets internalized once it reaches the cell [53]. The recurring pattern across studies is adaptation. Nanoparticles engineered not just to circulate through the bloodstream but also to recognize what they encounter, to bind when they find their target, and to respond appropriately within a living environment that keeps changing as the disease progresses [50,53,54].

Once targeting works, once the nanoparticle reaches the tumor, the next question follows naturally. When should the drug be released? How should it happen? [52,55,56]. Controlled release systems make nanomaterials responsive to their environment. They allow the drug to activate under specific conditions. Changes in pH, enzyme activity that differs between tumor and normal tissue, temperature shifts, and exposure to external light [55]. Smart nanotheranostic platforms take this further by combining diagnostic imaging with treatment. They create systems that both detect where cancer is and treat it in a coordinated way, almost simultaneously [56]. The logic behind this is deliberate. Avoid releasing the drug too early while it is still circulating. Maximize the concentration that builds up inside the tumor itself. Synchronize when the drug acts with what the biology of the tumor is doing at that moment. Release timing has a profound effect on how well the therapy works and how toxic it becomes to surrounding tissue [52]. Nanomaterials are increasingly built as responsive systems rather than static ones. They are capable of adapting their behavior to the tumor's biochemical environment, to what is happening around them, rather than just carrying drugs passively from one place to another [55,56].

Despite how sophisticated nanoparticle design has become, barriers remain when trying to bring these systems into actual clinical use [52-54]. Biocompatibility concerns arise. Questions about long-term toxicity. How the immune system might react to foreign nanoparticles. Uncertainty about where exactly these particles go once inside the body and how long they stay there [52]. Nanoparticles may gather in organs they were not intended for. The liver, the spleen, and other parts of the reticuloendothelial system. This raises concerns about chronic exposure over time, about how they eventually get cleared from the body, and whether that clearance is complete [53]. Manufacturing at scale introduces its own challenges. Making sure each batch is identical to the last. Getting through the layers of regulatory evaluation that any new therapeutic approach must pass [54]. The research does not dismiss these challenges or treat them as minor obstacles. It emphasizes the need for rigorous testing before any clinical use for standardized ways of profiling safety across different nanoparticle designs [52,53]. What becomes clear is that innovation alone does not guarantee adoption into clinical practice. Each advancement has to pass through multiple layers of biological testing, thorough assessment of how the drug moves through the body and how it acts in different tissues, and extensive regulatory scrutiny before it can be integrated into routine oncology care [52,54].

Looking ahead, nano-oncology feels less like a single technique or technology and more like a framework that keeps evolving, expanding into new areas [53,55,56]. The integration of nanotheranostics, combination therapies that use multiple mechanisms at once, and platforms designed to do several things simultaneously. All of this suggests a future in which diagnosis and treatment happen together at the nanoscale rather than as separate sequential steps [56]. Systems are being developed that are capable of imaging a tumor, delivering drugs to it, and monitoring the response all at the same time. Compressing what used to be multiple separate clinical steps into one unified structure [55]. It is also necessary to distinguish between established components of clinical oncology practice and approaches that remain largely experimental. A limited number of nanomedicine systems, including liposomal formulations of chemotherapeutic agents, are already approved and are now used routinely to enhance drug delivery and reduce toxicity in selected cancers [57]. These represent the more established side of the field, where safety and efficacy have been validated over time. However, many of the more advanced nanotechnologies reported in current research, particularly multifunctional nanotheranostic systems and highly engineered smart delivery systems, are still predominantly at the preclinical stage or in early-phase clinical trials [52,56]. While their potential is extensively studied, clinical adoption remains limited and inconsistent.

This distinction is relevant because the rate of scientific innovation frequently exceeds the pace of regulatory approval and clinical integration. Promising results obtained in laboratory or early translational studies do not always automatically translate into oncology practice, where safety, reproducibility, and long-term outcomes must be established prior to widespread implementation. It is also important to distinguish between nanotechnologies that are already part of clinical oncology practice and those that remain largely experimental. Some nanomedicine formulations, particularly liposomal drug delivery systems such as liposomal doxorubicin, have already received regulatory approval and are used clinically to improve drug distribution and reduce systemic toxicity in selected cancers [57]. These represent the more established side of nano-oncology, where therapeutic benefit and safety profiles have been evaluated over years of clinical use.

However, many of the more advanced nanotechnologies described in current literature, especially multifunctional nanotheranostic platforms and highly engineered smart delivery systems, remain predominantly within preclinical development or early-phase translational studies [52,55,56]. While experimental findings are promising, broader clinical implementation is still limited by challenges involving large-scale manufacturing, reproducibility, long-term toxicity evaluation and regulatory standardization [52,54]. This distinction matters because scientific innovation in nano-oncology often progresses faster than clinical integration. Results obtained under controlled laboratory conditions do not always translate directly into routine oncology practice, where therapies must demonstrate sustained safety, reproducibility, and measurable patient benefit before widespread adoption becomes feasible.

Nanotechnology continues expanding beyond just transporting chemotherapy drugs from one place to another. It is moving into gene editing, into modulating how the immune system responds to cancer, and into precision diagnostics that can detect disease earlier [53]. The field is moving toward convergence. Materials science, molecular oncology, bioengineering. All of these are interlacing into therapeutic systems that can adapt to what they encounter [56]. The direction of progress is incremental but persistent. Each advance builds on what came before. It reflects a broader shift happening in oncology toward interventions that are not only targeted to specific molecular features but structurally designed and engineered from the ground up to be responsive, adaptive, and precise [53,55,56].

Digital health, tele-oncology, and remote monitoring

Telemedicine in oncology did not emerge as a replacement for in-person care; it came as a response to fragmentation in treatment pathways and to gaps that arise when care spans time and distance [15,58]. Cancer care is rarely contained to a single moment or visit. It often requires frequent follow-ups; continuous monitoring of symptoms as they develop and change; and coordination among multiple specialists and departments who may not always communicate seamlessly with each other [15]. Digital health and telehealth models try to support continuity by bridging physical distance and by enabling structured communication between patients and the providers caring for them, even when they are not in the same room [15]. Proactive tele-oncology nursing triage models take this concept further. They use remote monitoring of data that patients generate themselves, data about symptoms, vital signs, and side effects, to detect when something is escalating early, before things worsen significantly enough to require emergency intervention [58]. The logic behind this approach is straightforward, yet it transforms how care actually happens in practice. Instead of waiting for a patient's condition to deteriorate and then responding reactively, clinicians can intervene earlier, preemptively, adjusting treatment or providing support before a crisis point is reached. What telemedicine does, when it works well, is compress the gap between when a symptom first appears and when clinical response occurs. It stabilizes the trajectory of care while preserving connection across distances that might be geographic, logistical, or both [15,48].

Remote monitoring becomes more tangible, more real, when wearable biosensors enter the picture. When technology moves from the clinic onto the patient's body [58,59]. Patients increasingly use mobile health technologies to record physiological parameters as they change throughout the day, to report symptoms in the moment they occur, to track treatment side effects in real time as they experience them rather than trying to recall them weeks later during a clinic visit [59]. Patients often value these systems when they perceive clear clinical benefit from using them, when there is transparency about what happens to their data once it is collected, particularly when the feedback loops feel responsive rather than one-directional and when they sense that someone is actually paying attention to what the data shows [59]. Structured triage models can convert this continuous stream of information into actionable clinical decisions that lead to tangible changes in care, rather than just accumulating data for its own sake [58]. Yet the human dimension remains central here. Data alone does not improve outcomes unless someone interprets it, places it in context, and understands what it means for this particular patient at this particular point in their treatment. Wearable technologies extend clinical observation beyond hospital walls, beyond the limited window of scheduled clinic visits. But their impact depends heavily on how well they integrate into organized care pathways and systems that know what to do with the information being generated continuously [58,59].

Electronic health records function as the structural backbone of digital oncology systems. They are more than documentation tools, more than just places where notes get stored [15]. Integrated digital platforms facilitate information sharing across different providers, reduce duplication of tests and procedures that waste time and resources, and support coordinated decision-making when multiple people are involved in a patient's care and need to stay aligned. Interoperability challenges and data fragmentation can undermine the goals of telehealth interventions, especially when systems fail to communicate efficiently and information becomes trapped in isolated databases that do not talk to one another. The promise of digital oncology lies in seamless integration. Clinical notes, laboratory results, imaging data, and outcomes that patients report about their quality of life and symptom burden all flow within a unified architecture rather than sitting in isolated silos that require manual searching and piecing together. Without that cohesion, without the pieces connecting smoothly, digital tools remain isolated enhancements scattered across the care pathway. They do not function as integrated components of a coordinated ecosystem that supports fluid decision-making and continuous care [15].

Patient engagement in digital oncology depends not merely on whether people have access to technology, own a smartphone, or have internet access at home. It depends on usability, on how intuitive the system feels, on trust [59,60]. Patients require clarity about what the platform does and why they should use it. They need assurance about privacy, about who sees their information, and how it might be used. They need an intuitive design that does not require extensive technical knowledge to navigate and does not add to the frustration of an already difficult situation [60]. Patients are more willing to adopt remote monitoring tools when they perceive that the system is personalized to them specifically and when the feedback they receive feels meaningful and relevant, rather than generic automated messages that could apply to anyone [59]. Engagement is fundamentally relational. It grows when platforms reflect what patients actually experience in their daily lives and when they align with expectations of responsiveness from the care team, using the technology feels like it strengthens rather than replaces human connection [60]. Mobile health technologies can empower patients to track their own symptoms, access information when they need it rather than waiting for the next appointment, and communicate with providers outside of scheduled visits when something concerns them. But sustained use over time requires systems that feel supportive rather than burdensome, that add genuine value to the patient's experience rather than creating extra work or causing anxiety [59,60].

The expansion of tele-oncology raises a question that cannot be avoided, and that becomes more pressing as these systems become more central to how care is delivered: who benefits from this and who is left behind [59,60]? Digital divides exist in telehealth accessibility, and they are not small or easily bridged. Disparities are linked to socioeconomic status, geographic location, whether broadband internet is available and affordable in a particular area, how familiar and comfortable someone is with technology, and whether they have devices that can run the necessary applications [61]. Patient expectations and digital readiness vary significantly across populations, shaped by education, by prior exposure to technology, and by cultural factors that influence how people relate to digital health tools [60]. While digital oncology promises a broader reach, the ability to extend care to more people in more places, including rural areas or underserved communities, its implementation can inadvertently widen inequities if infrastructure gaps and education gaps are not addressed directly and deliberately [61]. The trajectory forward requires more than just deploying technology. It requires deliberate policy decisions about where resources go, inclusive design that considers diverse user needs from the beginning, rather than retrofitting accessibility later, and targeted support mechanisms that help people who face barriers to access or use. The goal is to ensure that technological advancement translates into equitable care delivery rather than creating stratified access where only some populations benefit while others fall further behind, excluded by the very systems meant to expand reach [60,61].

Future technological horizons in oncology management

Drug discovery in oncology has traditionally been slow. Years were spent on molecular screening, testing one hypothesis at a time, watching compounds fail at various stages before anything reached patients [62]. Artificial intelligence changes the scale of what can be explored. Particularly when examined alongside quantum computing frameworks, it becomes possible to model molecular interactions at levels of complexity that classical computing systems cannot handle. The depth is computational. Vast chemical spaces can be analyzed, spaces that would take decades to explore physically in a laboratory. Binding affinities between molecules can be predicted before any synthesis happens. Molecular configurations can be optimized on a computer before committing resources to actually making them. But moving from what an algorithm predicts to a compound that works clinically remains complicated. Predictions need verification, models need testing against biological reality. What works in a simulation does not always work in a living system. Laboratory science is not being replaced here. It is being accelerated. AI explores possibilities, eliminates unlikely candidates early, and narrows the field before empirical testing begins with the options most likely to succeed. Current applications of quantum computing in oncology are largely experimental and have not been adopted clinically. Although computational modeling may accelerate molecular screening and drug optimization, most proposed healthcare applications are still in early developmental or pilot stages. Significant technical limitations must be addressed before routine integration into oncology drug discovery pipelines becomes feasible [62].

Genome engineering enters oncology with an ambition. To intervene at the level of the genetic mutations that drive cancer, to target them directly at their source. This kind of manipulation sits within a broader computational transformation happening across healthcare. Editing a genome is not just a molecular procedure anymore, not something done in isolation. Predictive analytics now inform the process. They assess off-target risks, model how different pathways might interact, and try to anticipate what might happen systemically before any editing takes place. Cancer's genetic architecture is intricate. Not a single mutation but networks of alterations interacting in ways that are difficult to predict. Intervening precisely requires modeling that anticipates ripple effects that consider what might change downstream when one gene is altered [62]. Clinical use depends on this computational layer. It refines where to target, helps identify the safest approach, and works to avoid unintended consequences before they appear in actual patients [50]. Although genome engineering technologies such as CRISPR-Cas have advanced considerably, their broader clinical use in oncology is limited by concerns over off-target effects, delivery efficiency, immune reactions, long-term genomic stability, and ethical oversight. As a result, many genome editing strategies for cancer management are in preclinical development or early translational evaluation rather than widespread clinical application. Genome engineering becomes part of something larger. Molecular tools working alongside advanced analytics rather than functioning separately [62].

A digital twin is a computational representation of a patient or tumor. The concept sounds abstract at first, but it is being developed as something practical, something that might be used clinically [63-65]. A digital twin pulls together multiple streams of information. Imaging data showing where the tumor is and how it has changed. Genomic profiles revealing mutations. Physiological measurements track how the body is responding. Records of past treatments and how they worked. All of this aggregates into a dynamic model [63,64]. The model can simulate what might happen if different treatments are applied. It tests interventions virtually before they happen in practice, projects that approach might work best for this particular case [64]. Predictive modeling estimates tumor response trajectories. It refines strategies beyond standardized protocols that treat every patient with the same diagnosis identically [65]. The system does not stay fixed; it updates continuously. As treatment progresses and new patient data come in, the model adjusts. It recalibrates based on what is actually happening rather than what was initially predicted. The architecture mirrors cancer itself, adaptive and constantly shifting as new information arrives [63-65]. However, most digital twin applications in oncology currently remain within translational research and early feasibility studies rather than routine clinical practice. Existing models have shown potential in areas such as radiotherapy planning and treatment-response simulation, but broader validation across diverse patient populations and real-world oncology settings remains limited [63-66]. Still, most of this work remains in translational research rather than being used in oncology practice. Some digital twin models have made it into clinical settings and early feasibility studies, especially for radiotherapy planning and predicting treatment response [66]. But broader validation across diverse patient populations is still limited. A key issue is data. Many of these systems rely on highly integrated datasets that are difficult to standardize across hospitals, and keeping patient models updated in real time requires substantial computational infrastructure [64,66]. There is also the question of clinical trust. Before these systems can guide treatment decisions, clinicians need to see that the predictions are reliable outside controlled research environments and across the variability seen in everyday oncology care [66]. Right now, digital twins exist somewhere between emerging clinical tools and evolving conceptual frameworks. The potential is clear, but integrating them into routine practice is happening gradually, not all at once. 

Oncology is generating more data than it used to. More genomic information, more imaging, more longitudinal records tracking patients over the years. Protecting that information becomes necessary as it moves across institutions [62]. Blockchain architectures offer a different approach from traditional centralized databases. They are decentralized, tamper-resistant, and designed to manage sensitive healthcare data in a way that reduces vulnerability. Genomic datasets need secure exchange. Imaging repositories need to move between institutions without compromising integrity or privacy. Treatment records need protection from unauthorized access or manipulation. Blockchain systems use distributed ledger frameworks. They track who accesses data and when. They preserve immutability, making it difficult to alter records retroactively. They reduce reliance on single centralized authorities that can become points of failure. But implementation faces constraints. Scalability becomes an issue when large volumes of data need to move quickly. Making blockchain systems work with existing infrastructure is not straightforward. Regulatory frameworks are still being developed in many places [62].

For now, most blockchain applications in oncology are still at the exploratory stage rather than being fully integrated into routine clinical systems. Pilot projects and research platforms have shown that decentralized architectures could help with secure genomic data exchange, managing patient consent, and linking data across institutions [67]. But actual use in hospitals remains rare. A big part of the problem is that existing hospital infrastructures are often built around centralized electronic records, and shifting that to a blockchain framework usually requires major structural changes. There are also questions around scalability, energy demand, governance, and whether these systems could keep up in real-time clinical settings where fast data access matters. The technology continues to attract interest because of its security potential. In practice, though, integration into oncology workflows is moving slowly, mostly through experimental and early translational projects rather than widespread clinical use. Wider adoption is hindered by several factors, including compatibility challenges with existing centralized electronic health record systems, concerns about scalability and energy consumption, uncertainties in governance and regulatory oversight, and doubts regarding whether blockchain infrastructures can meet the speed and efficiency demands of real-time clinical oncology workflows [67].

Advances in computational oncology require matching advances in security. Systems that sustain trust while enabling the data sharing that analytics depend on. Systems that protect patients without preventing the flow of information that makes advanced care possible [62]. These innovations are beginning to align. They are starting to connect rather than remaining isolated developments [63,65]. Personalized medicine bridges technological innovation and clinical application. It connects what can be done computationally to what is actually implemented when caring for patients [65]. Digital twins function as platforms that bring together diverse data streams and information that might otherwise stay fragmented across different systems [63]. Biotechnology generates molecular insights; it reveals what is happening genetically and at the cellular level. Informatics structures that information, predicts outcomes, and makes sense of complexity that would be difficult to process manually. Clinical practice interprets the outputs. It considers not just what the technology suggests but what makes sense for this person, in this context, with these particular circumstances. Oncology management is shifting away from isolated breakthroughs and toward coordinated systems in which different components work together [63,65]. Technologies communicate. Predictive models refine their strategies as they process more cases. At the end of the day, these new technologies are meant to support clinical judgment, not take its place. The systems are only as good as the quality of the data they are trained on. Hence, when it comes to deciding on treatment, it still comes down to the clinicians to interpret the results [62-67]. Clinicians apply judgment within increasingly data-informed environments, balancing what systems recommend with what experience suggests and what the specific situation calls for. The landscape is complex, but it has shape. Dynamic interaction between biological manipulation, computational modeling, and care at the bedside. Each informs the others. Each is necessary but not sufficient on its own [63,65].

## Conclusions

Today’s cancer management transcends isolated specialties, reflecting a deepened understanding of malignancy at the molecular level. This knowledge enhances diagnosis, therapy design, and care delivery, incorporating precision imaging, liquid biopsies, engineered immune responses, nanoscale drug systems, and robotic platforms. These elements are now integral to treatment. Digital infrastructures facilitate continuity of care beyond hospital walls, embedding data into decision-making and enabling predictive models of disease and therapeutic outcomes. Yet moving from a promising technology to something used routinely in clinical practice is rarely straightforward. Regulatory approval is demanding, digital systems often don’t integrate well with one another, and clinicians are still learning to adapt their workflows to incorporate new technical skills alongside the clinical judgment they rely on.

Access is uneven as well. Hospitals with advanced infrastructure can adopt digital oncology tools more quickly, but those with fewer resources are limited by funding, unreliable connectivity, limited staff training, and basic access to the technology itself. These gaps shape how consistently innovation can be integrated into everyday patient care. Oncology is shifting to an anticipatory model that addresses potential developments proactively. The relationship among biological insight, engineering, and data improves clinical judgment and fosters a cohesive, patient-centered oncology framework that prioritizes both innovation and the integration of equitable care.
